# Transdiaphragmatic-pericardial Hernia: Case Report of an Unusual Condition Managed by Utilization of a Robotic Surgical System

**DOI:** 10.7759/cureus.7689

**Published:** 2020-04-16

**Authors:** Anupam K Gupta, Mridul Pansari, Slee Yi, Thomas Genuit, Ariel Rodriguez

**Affiliations:** 1 Surgery, Charles E. Schmidt College of Medicine, Florida Atlantic University, Boca Raton, USA; 2 Surgery, Florida Atlantic University, Boca Raton, USA

**Keywords:** diaphragmatic hernia, transdiaphragmatic hernia, iatrogenic hernia, robotic hernia

## Abstract

Transdiaphragmatic hernia via the central tendon in the pericardium is a rare entity. Most often, these hernias are acquired and related to the iatrogenic creation of a transdiaphragmatic-pericardial defect. We would like to present a case of a 62-year-old male who presented with acute chest pain and was diagnosed to have a transdiaphragmatic-pericardial hernia on computed tomography. He has a history of cardiac intervention via an abdominal approach 12 years earlier. A segment of jejunum was found strangulated in the defect. We used DaVinci Xi® (Intuitive Surgical, Sunnyvale, CA) robot and were able to successfully reduce the hernia, repair the defect, and resect a short segment of jejunum which was non-viable. The utilization of a minimally invasive approach may be feasible, and the use of a robotic surgical system may enhance the surgeon’s ability to repair the defect.

## Introduction

Atrial fibrillation is one of the most common arrhythmias. A variety of cardiac procedures are used to treat atrial fibrillation. Some of these procedures require a subxiphoid transdiaphragmatic approach [1,2]. In this process, the pericardium needs to be opened to approach the heart. Following this approach, the pericardium is typically not closed after and can lead to a potential defect [1-3]. This defect can lead to a rare complication called transdiaphragmatic-pericardial hernia (TDPH). 

Diaphragmatic hernias can be acquired or congenital [4,5]. Acquired TDPH is unusual and usually secondary to a prior iatrogenic procedure [4-9].

We would like to present an unusual case of bowel strangulation in a TDPH which occurred secondary to a prior transdiaphragmatic cardiac procedure for atrial fibrillation.

## Case presentation

A 62-year-old male patient presented to the emergency room with an acute complaint of chest pain. The patient’s past medical and surgical history were significant for hypertension, hyperlipidemia, and coronary artery disease. The patient had undergone percutaneous cardiac stent placement and a cardiac ablation procedure for atrial fibrillation, through a subxiphoid transdiaphragmatic approach, 12 years prior.

**Figure 1:**
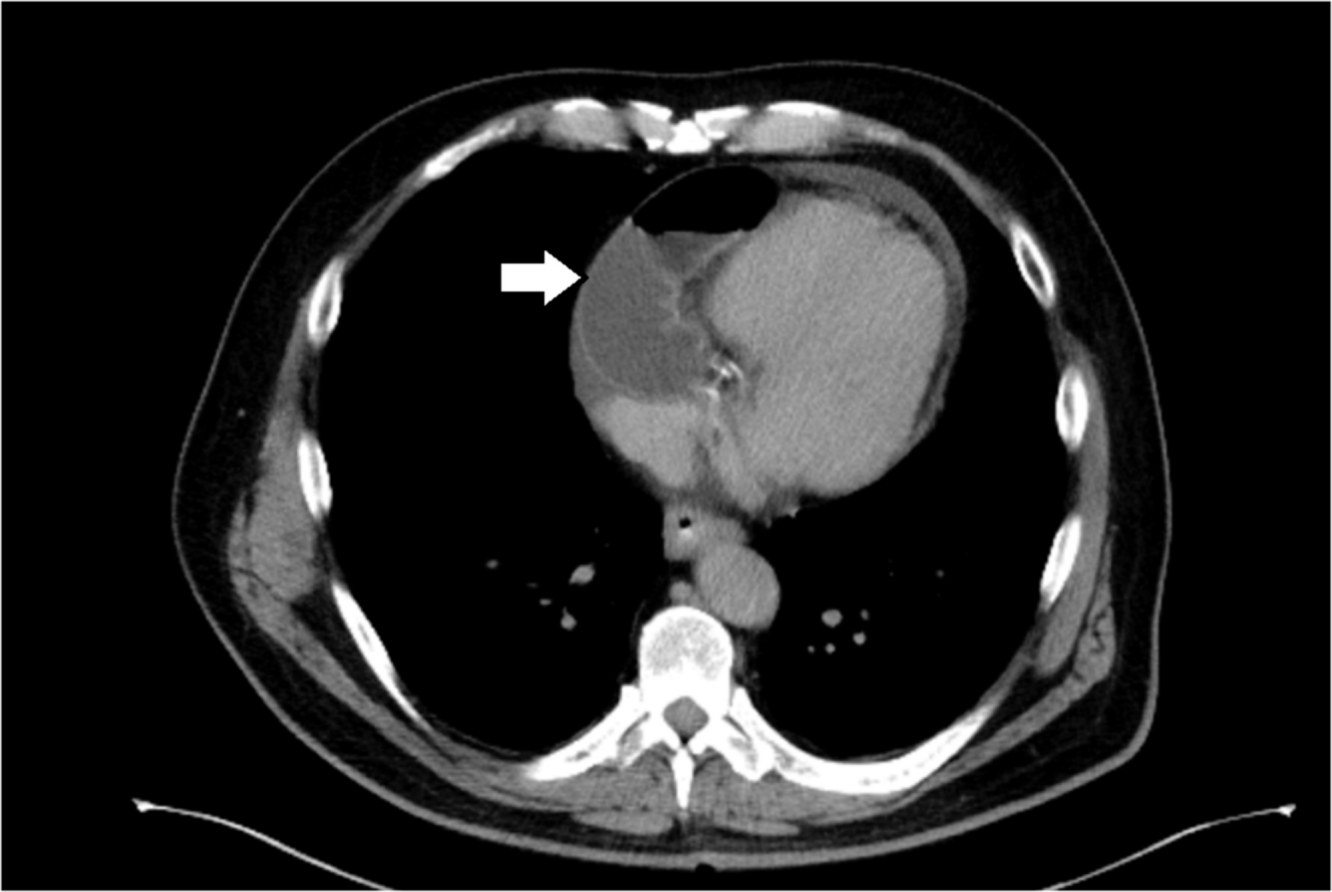
Computed tomography demonstrating a small bowel loop next to the heart in the pericardial sac

On presentation, the patient complained of acute-onset substernal chest pain, with radiation to the left arm, associated with one episode of non-projectile bilious vomiting, leading to the evaluation and exclusion of acute cardiac ischemia. The patient’s vital signs were within normal limits. On physical examination, the patient’s chest examination was within normal limits, and the abdomen was soft, not distended, and without evidence of peritoneal signs. His initial workup included an electrocardiogram and cardiac enzymes, which did not reveal any evidence of myocardial infarction. Laboratory evaluation, including a comprehensive metabolic panel and complete blood count, also demonstrated values within normal limits. CT of the chest and abdomen with and without intravenous contrast revealed the presence of a transdiaphragmatic hernia with apparent small bowel contents adjacent to the heart and within the pericardium (Figure 1).

**Figure 2:**
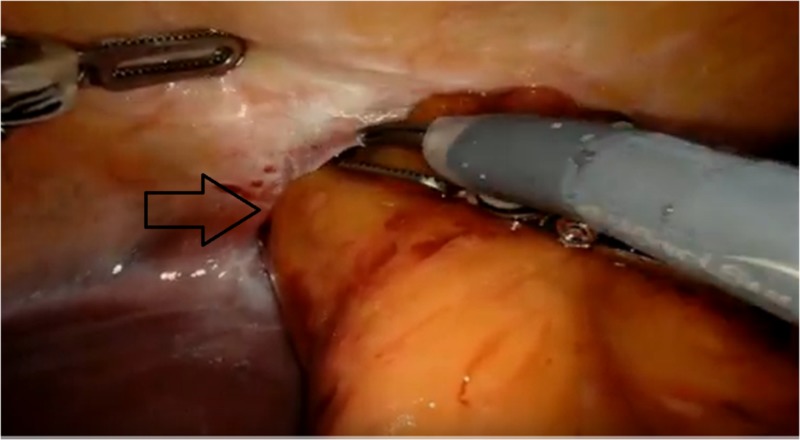
Intraoperative view showing the transdiaphragmatic hernia defect with contents entering the pericardial space

Given the patient’s symptoms, and concerns for possible bowel incarceration, the patient was taken emergently for robotic-assisted surgery (Intuitive da Vinci XI® robotic system, Intuitive Surgical, Sunnyvale, CA). Four 8-mm ports were placed, two in the right upper quadrant (anterior axillary line), one port in the left upper quadrant (anterior axillary), and one video port in the supraumbilical region. Upon exploration, a loop of small bowel could be seen entering the chest via an anterior midline diaphragmatic defect, which measured approximately 6 x 4 cm (Figure 2).

Attempts at reducing the herniated bowel were initially unsuccessful due to adhesions and the apparent incarceration of the small bowel loop. After extending the defect laterally in either direction, reduction of the herniated bowel became possible. On inspection, the loop was consistent with the proximal jejunum and demonstrated a 5-cm segment of transmural ischemia. The ischemic segment was resected, and the bowel continuity restored with a stapled side-to-side, functional end-to-end jejunojejunostomy. The pericardium was inspected and carefully irrigated; there was no evidence of intrapericardial inflammation or infection. The pericardial defect was closed primarily using a continuous 2.0 polydioxanone (PDS) suture and the diaphragmatic defect was then closed, using a poly-4-hydroxybutyrate (P4HB) surgical mesh with a hydrogel barrier (Phasix-ST®, C.R. Bard, Inc., Murray Hill, NJ). The mesh was secured to the diaphragm circumferentially with interrupted 2.0 polyglactin (Vicryl) sutures.

Postoperatively, the patient’s recovery was uneventful; he was started on a liquid diet on day 1 and advanced to a regular diet by day 3. The patient was discharged on postoperative day 3 and at the two-week follow up visit, the patient was doing well without any signs of complications.

## Discussion

TDPH is a rare condition. It can occur due to diaphragmatic-pericardial defects that are usually the sequelae of traumatic injuries or the iatrogenic creation of a defect [3-6]. Iatrogenic defects most often follow coronary bypass surgery, particularly when using a right gastroepiploic artery graft, following subxiphoid procedures, including pericardial window or procedures for atrial fibrillation or after esophagectomy [1-3]. The herniation of abdominal contents is a result of pressure differences between the abdomen and thoracic cavity and may involve any of the abdominal hollow viscus organs [7-9]

The clinical presentation of TDPH may be variable. Patients can present with symptoms of chest pain, mimicking cardiac ischemia or abdominal pain, indigestion and nausea with or without vomiting, or can be asymptomatic. This makes the clinical diagnosis at times difficult [4-9]. The physical examination is often not helpful but can include the findings of bowel sounds over the mid-chest, decreased breath sounds over the left anterior lung fields, and the absence of a cardiac apex impulse [6-11]. Imaging is key in the diagnosis of TDPH: plain-film chest radiography may demonstrate an enlarged cardiac silhouette and/or what appears to be a pneumopericardium, but is often not diagnostic [8]. CT of the chest and abdomen reveals the herniated viscera within the pericardium [4]. 

Once the diagnosis is made, urgent or emergent surgical repair is indicated to prevent secondary complications of bowel incarceration, strangulation, or the physiologic effects of cardiac tamponade. Both open and laparoscopic surgical repairs have been reported for TDPH, using both abdominal and thoracic approaches [4-9,12]. When utilizing a thoracic approach, individual case reports have described using a median sternotomy, but most successfully utilized a left anterior lateral thoracotomy or minimally invasive approach, avoiding the added morbidity associated with median sternotomy [4,5,8]. 

Primary repair of the diaphragmatic defect with non-absorbable or slowly absorbable monofilament suture may be feasible for smaller defects; a mesh repair is most often used for larger defects. Most surgeons seem to use composite mesh; however, there is no clear consensus on the type of mesh most appropriate for diaphragmatic hernia repair [3-13]. Robotic-assisted surgical approaches are becoming increasingly popular, as they can provide added ability for visualization and dexterity while mobilizing the herniated contents and repairing the TDPH defect [14]. 

In the published literature, pericardial defects after cardiac interventions are most often left unclosed [1,2]. While thousands of these procedures are performed annually all over the world, the true incidence of TDPH is unknown [9,10,13].

## Conclusions

TDPH is a rare diagnosis that requires urgent or emergent surgical therapy. A minimally invasive use of robotic-assisted surgery may permit better visualization, manipulation of hernia contents, and easier repair of the defect.
